# Systemic pentraxin-3 levels reflect vascular enhancement and progression in Takayasu arteritis

**DOI:** 10.1186/s13075-014-0479-z

**Published:** 2014-11-14

**Authors:** Enrico Tombetti, Maria Chiara Di Chio, Silvia Sartorelli, Maurizio Papa, Annalaura Salerno, Barbara Bottazzi, Enrica Paola Bozzolo, Marta Greco, Patrizia Rovere-Querini, Elena Baldissera, Alessandro Del Maschio, Alberto Mantovani, Francesco De Cobelli, Maria Grazia Sabbadini, Angelo A Manfredi

**Affiliations:** Department of Medicine, IRCCS San Raffaele Scientific Institute, via Olgettina 60, 20132 Milano, Italy; Vita-Salute San Raffaele University, 20132 Milano, Italy; Department of Radiology, IRCCS San Raffaele Scientific Institute, via Olgettina 58, Milano, 20132 Italy; Istituto Clinico Humanitas, Istituto di Ricovero e Cura a Carattere Scientifico, Rozzano, 20089 Italy; Dipartimento di Medicina Traslazionale, Università di Milano, Rozzano, 20089 Italy

## Abstract

**Introduction:**

Progression of arterial involvement is often observed in patients with Takayasu arteritis (TA) thought to be in remission. This reflects the failure of currently used biomarkers and activity criteria to detect smouldering inflammation occurring within arterial wall. Pentraxin-3 (PTX3) is a soluble pattern recognition receptor produced at sites of inflammation and could reveal systemic as well as localized inflammatory processes. We verified whether the blood concentrations of PTX3 and of C-reactive protein (CRP) in patients with Takayasu arteritis (TA) might reflect vascular wall involvement, as assessed by signal enhancement after contrast media administration, and the progression of arterial involvement.

**Methods:**

A cross-sectional single-centre study was carried out on 42 patients with TA that comprised assessment of PTX3, of CRP and erythrocyte sedimentation velocity (ESR). In total, 20 healthy controls and 20 patients with Systemic Lupus Erythematous (SLE) served as controls. Vascular imaging was carried out by magnetic resonance angiography, doppler ultrasonography and computed tomography angiography.

**Results:**

Patients with TA and SLE had higher plasmatic PTX3 and CRP concentrations than healthy controls (*P* = 0.009 and 0.017, respectively). PTX3 levels did not correlate with those of CRP. Patients with active systemic TA had significantly higher concentrations of CRP but similar levels of PTX3 than patients with quiescent disease. In contrast, patients with vascular inflammation detectable at imaging had higher PTX3 concentrations (*P* = 0.016) than those in which vessel inflammation was not evident, while CRP levels were similar. The concentration of PTX3 but not that of CRP was significantly higher in TA patients with worsening arterial lesions that were not receiving antagonists of tumor necrosis factor-α or interleukin-6.

**Conclusions:**

Arterial inflammation and progression of vascular involvement influence plasma PTX3 levels in TA, while levels of CRP accurately reflect the burden of systemic inflammation. These results support the contention that PTX3 reflects different aspects of inflammation than CRP and might represent a biomarker of actual arteritis in TA.

## Introduction

Takayasu arteritis (TA) is a rare, idiopathic, chronic-relapsing inflammatory disease, typically affecting young women, characterized by considerable morbidity and mortality [1-4]. Inflammation primarily localizes in the large arteries, such as the aorta, the pulmonary artery and their major branches. Arterial inflammation results in thickening of the vessel wall with eventual stenosis/occlusion, or in vessel dilatation and aneurysm formation [1,4,5]. The lack of accepted and reliable criteria for disease activity has limited clinical management of patients as well as clinical research. Moreover, commonly used biomarkers do not accurately discriminate active and inactive disease [1,6]. Erythrocyte sedimentation rate (ESR) and C-reactive protein (CRP) levels, which are elevated during active phases of the disease in almost half of the cases, are unreliable markers of disease activity, vascular inflammation and progression [4,5,7]. Multi-item activity criteria have been proposed, such as the National Institutes of Health (NIH) criteria [5]. However they lack accuracy and patients considered inactive often undergo vascular progression [5,7], suggesting that these criteria do not take into account smouldering inflammation occurring within vessel walls. Conversely, not all TA patients experiencing systemic inflammatory flares undergo anatomical progression of the vascular involvement [8].

Aortic regurgitation, hypertension, retinopathy and aneurysms, all contribute to the mortality of patients with TA [9]. They depend on the characteristics of the inflammatory involvement and of the remodelling of arteries [10,11]. Therefore to prevent vascular progression is a reasonable hard outcome and a therapeutic goal in TA. The availability of reliable imaging tools and of biomarkers for the assessment of vascular involvement is thus essential for the management of patients with TA.

Pentraxin-3 (PTX3) is a long pentraxin produced at sites of inflammation in response to microbial or sterile stimuli [12,13]. PTX3 has a non-redundant role in the protective response against selected pathogens and regulates various events implicated in TA pathogenesis, including innate immunity and inflammation, extracellular matrix deposition, tissue remodelling and vascular homeostasis [12-14]. PTX3 has been proposed to reflect disease activity in small vessel vasculitis [15,16] and more recently in giant cell arteritis [17] and TA [18-20]. Here we explore the link between PTX3 plasma levels, vascular inflammation and vascular progression.

## Methods

### Study sample

**Figure 1:**
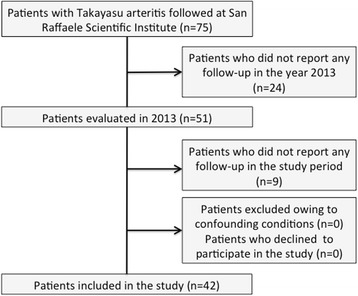
**Study flow diagram.**

Consecutive patients with TA (n = 51) have been evaluated in 2013 at San Raffaele Scientific Institute in Milan. All patients fulfilled the American College of Rheumatology (ACR) criteria for TA [21] and had been extensively evaluated to exclude conditions that could mimic the disease such as Behçet disease, Cogan syndrome, Kawasaki disease, giant cell arteritis, Marfan syndrome, Ehler-Danlos syndrome, neurofibromatosis and infective aortitis. We performed a cross-sectional analysis between April and August 2013, excluding nine patients who did not attend a visit in this period. The final sample comprised 42 patients (Figure 1): 20 healthy blood donors and 20 consecutive patients fulfilling the ACR criteria for systemic lupus erythematous (SLE) who visited at our outpatient clinic in May 2013 served as controls. Subjects in control groups and patients with TA had similar age and sex distribution. All subjects gave written informed consent for participation in the study and the Institutional Review Board (*Comitato Etico dell’Ospedale San Raffaele*, *Milano*) approved storage of the plasma in the biobank.

### Biomarkers

Plasma concentrations of PTX3 were measured by a sandwich enzyme-linked immunosorbent assay, as previously described [22]. This assay is highly sensitive and specific [22,23]. No cross-reactions were observed with other pentraxins, including CRP and serum amyloid P. CRP was measured with a latex-enhanced immunoturbidimetric assay (ADVIA Chemistry System, Bayer Healthcare AG, Leverkusen, Germany). ESR was evaluated by the Westergren method. There were no samples missing for PTX3 and CRP assessment but three samples were missing for ESR. Samples were analysed in a random order and in a blinded manner. Systemic activity of TA was evaluated according to NIH criteria [5]. Complement levels and anti-DNA antibody titres of patients with SLE were also assessed and disease activity assessed using the validated index systemic lupus erythematosus disease activity index (SLEDAI).

### Imaging

All patients with TA were initially evaluated by magnetic resonance angiography (MRA) and vascular ultrasonography (US). Two patients had contraindications to MRA and computed tomography angiography (CTA) was used instead. MRA was performed with a 1.5-T magnetic resonance whole body scanner (Achieva Nova Master; Philips Medical System, Best, The Netherlands) with phased-array head and neck or phased-array thoracic dedicated coils. Morphologic sequences (PD Black Blood TSE: field of view (FOV) 260 × 152; acquisition (Acq) matrix = 260 × 264; reconstruction (Recon) matrix = 528; echo time/repetition time (TE/TR) = 20/2 beats; ECG triggered; in expiratory breath-hold of approximately 10 sec) were performed to evaluate vessel wall thickening. First pass MRA dynamic sequences targeted on the thoraco-abdominal aorta and supra-aortic trunks (FOV 450 × 390; Acq matrix = 352 × 200; Recon matrix = 670; Acq voxel = 1.28/1.90/3.00; Recon voxel = 0.67/0.67/1.5; slice thickness = 1.5 mm; TR/TE/α = 4.6/1.35/40; Acq time = 21 sec) were performed during contrast media infusion (Gadovist®: 1 nmol/ml Bayer-Shering Pharma, Berlin, Germany). High resolution (HR) sequences were performed before and after contrast administration (Coronal HR 3D FFE: FOV 360 × 276; Acq matrix = 684 × 521; Recon matrix = 880; slice thickness = 0.8 mm; TR/TE/FA = 6.3/2.1/20; Acq time = 3 minutes and 28 seconds). These sequences allow the evaluation of vessel wall thickness up to 1 mm. We were actively looking for approaches to evaluate vessel wall enhancement because Gadofosveset Trisodium was retired [24]: the evaluation of vessel wall enhancement was given to radiologist interpretation. CTA was performed with a Brilliance CT 64-channel scanner (Philips Medical System®). The evaluation of vessel wall enhancement was given to radiologist interpretation because no cutoff value has been established with this technique. CT was performed with 130 ml of Ultravist 370 (Bayer-Shering Pharma, Berlin, Germany) administration at a rate of 5 ml/sec. US was performed with the iU22 Matrix Ultrasound system (Philips Medical System®). US and CTA evaluations assessed the carotid, subclavian, abdominal and femoral regions, while MRA focused on thoraco-abdominal or cervico-thoracic regions according to disease involvement. Two radiologists, blinded to the clinical status of patients, performed MRA, CTA and US examinations. Each lesion was evaluated independently. Variables evaluated for each lesion at MRA and CTA included width, length, residual lumen and contrast enhancement. On US, lesion width, residual lumen, blood-flow peak systolic rate and the presence of the halo sign were assessed. Imaging examinations are repeated yearly or in the case of relapses, until disease enters the tardive phase, with clinical and morphological stability for at least 3 to 5 years.

To evaluate vascular progression we relied on clinical records of US examinations and on review of imaging data for MRA and CTA. Progression was defined as appearance of novel lesions or as increase in width and/or length and/or percentage of luminal stenosis when established lesions were evaluated. Imaging evaluation of vascular progression was carried out yearly or when there was disease relapse. Atherosclerotic lesions were identified based on suggestive localization at arterial branches, eccentric appearance of the lesions, short and focal lesions, inhomogeneous content and irregular luminal border on US, the presence of parietal calcifications on the luminal side of the lesion and absence of halo signs or adventitial thickening on US.

### Statistical analysis

We express plasma PTX3 and CRP levels, ESR and other scalar variables as medians values and ranges. Mean values are also shown. The Mann-Whitney *U*-test was used to compare biomarkers between patients with TA and controls or between various subgroups of patients with TA stratified according to the presence or the absence of wall enhancement, of vascular progression, of active disease and history of aneurysms. The Kruskal-Wallis test was used to compare values of biomarkers in the various classes of arterial involvement [25]. We used the Spearman rank correlation coefficient to describe the correlation between PTX3 and CRP and between PTX3 and ESR. A two-tailed *P*-value less than 0.05 was considered to represent a statistically significant difference. All statistical analysis was performed with IBR® SPSS® statistics, version 20 (IBM corporation, Armonk, NY, USA).

## Results

### Patient characteristics

Table 1 summarizes the demographic and clinical characteristics, ESR, CRP and PTX3 levels of the 42 patients (39 women and 3 men) with TA. Median age at the study entry was 46 years (range 23 to 66 years) and median age at disease onset was 30 years (range 17 to 56 years). Demographic features were similar in the control groups (20 healthy women with a median age of 37 years (range 24 to 57 years), 18 women and 2 men with SLE and a median age of 44 years (range 21 to 61 years)). Most TA patients had widespread diffuse arterial involvement (angiographic class II or V). Sixteen patients (38%) had arterial aneurysms and 38 patients (90%) were on treatment: 30 patients received steroids, 30 immunosuppressive agents (12 azathioprine, 11 methotrexate, 4 mofetil mycophenolate, 2 sirolimus, 1 cyclophosphamide), 16 TNF-blockers, 2 tocilizumab and 1 rituximab. Twelve patients (29%) fulfilled the NIH criteria for active TA. Arterial wall enhancement was detectable in 16% (5/30, of whom 3 had active disease) and vascular progression in 22% (9/40) of the patients for which recent previous imaging data were available (Table 1).

**Table 1:** **Characteristics of the patients with Takayasu arteritis (n = 42)**

**Variables**	**Value**
**Qualitative variables, number (percent)**	
Sex, female:male	39:3
Class of vascular involvement	
1	4 (10%)
2A	4 (10%)
2B	3 (7%)
3	1 (2%)
4	0
5	30 (71%)
Coronary involvement	6 (14%)
Pulmonary artery involvement	13 (31%)
Aneurysms	16 (38%)
Steroids	30 (71%)
Immunosuppressive therapy	30 (71%)
Azathioprine	12 (29%)
Methotrexate	11 (26%)
Mycophenolate	4 (10%)
Sirolimus	2 (5%)
Cyclophosphamide	1 (2%)
Biologic therapy	19 (45%)
TNF-blockers	16 (38%)
Tocilizumab	2 (5%)
Rituximab	1 (2%)
Active disease (National Institutes of Health criteria)	12 (29%)
Vascular enhancement (n = 30)	5 (16%)
Vascular progression (n = 40)	9 (22%)
**Scalar variables, mean, median (range)**	
Age, years	45, 46 (23 to 66)
Age at disease onset, years	33, 30 (17 to 56)
Disease duration, years	12, 10 (0 to 34)
Prednisone (PDN) dose, mg/day (n = 30)	9.2, 5 (3 to 35)
Erythrocyte sedimentation rate, mm/h	22, 15 (1 to 78)
Serum C-reactive protein, mg/l	6.2, 2.3 (0.1 to 40)
Serum Pentraxin-3, ng/ml	8.4, 5.5 (1.3 to 55)

### Elevated plasma PTX3 concentrations reflect ongoing vascular inflammation in TA patients

**Figure 2:**
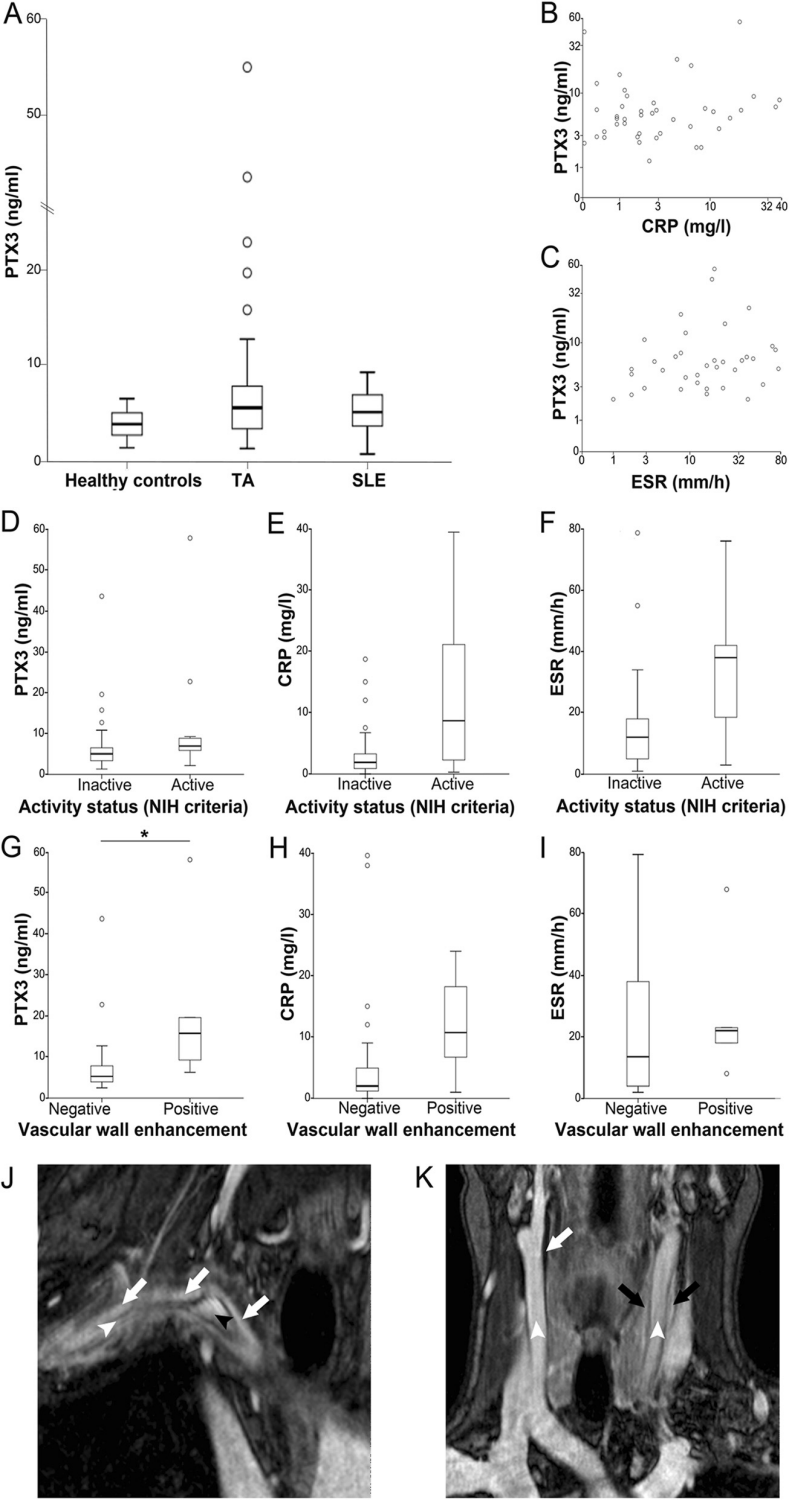
**Pentraxin-3 (PTX3) levels reflect ongoing vascular inflammation in Takayasu arteritis (TA). (A)** Plasma concentrations of PTX3 in patients with TA and matched systemic lupus erythematosus (SLE) patients or healthy controls. **(B,** **C)** Plasma concentrations of PTX3 in patients with TA do not correlate in patients with C-reactive protein (CRP) or erythrocyte sedimentation rate (ESR) levels. **(D-** **F)** CRP and ESR levels, but not PTX3, identify ongoing systemic inflammation in patients with TA, as assessed using the National Institutes of Health (NIH) criteria for active and inactive disease. **(G-** **I)** PTX3, CRP and ESR levels stratified on the basis of vascular wall enhancement. PTX3 is a more sensitive maker of vascular inflammation, based on the presence of vascular wall enhancement after contrast medium infusion, than CRP of ESR. **(J)** Representative images of magnetic resonance angiography (MRA) assessment of vascular inflammation in a patient with TA, with vivid enhancement in the thickened arterial wall of the anonym and subclavian arteries (white arrows); patent lumen of the anonym artery (black arrowhead); sub-occluded lumen of the subclavian artery (white arrowhead). **(K)** MRA image of a patient with TA with thickened left carotid artery with enhancement (black arrows) and thickened right carotid artery without enhancement (white arrows); white arrowheads indicate patent arterial lumen.

The PTX3 concentrations in the blood of patients with TA (median 5.5 ng/ml, range 1.3 to 55 ng/ml) and SLE (median 5.1 ng/ml, range 0.7 to 9.2 ng/ml) were significantly higher than those in healthy controls (median 3.9 ng/ml, range 1.4 to 6.5 ng/ml, *P* = 0.009 and 0.017 respectively). The PTX3 concentrations were similar in patients with TA and SLE (Figure 2A). Classes of arterial involvement or aneurysmal TA did not influence PTX3 and CRP concentrations or ESR (Table 2). PTX3 levels in TA patients did not correlate with CRP levels or with ESR (Figure 2B and C). PTX3 levels were similar in patients identified according to the NIH criteria as having active or inactive TA. In contrast, CRP levels and ESR were higher in patients deemed to have active TA according to the NIH criteria (respectively *P* = 0.013 and *P* = 0.011, Figure 2D-F). The results suggest that events other than systemic inflammation, which is well identified by NIH criteria and for which CRP and ESR are effective biomarkers, are responsible for PTX3 elevation in patients with TA.

**Table 2:** **ESR, and levels of CRP and PTX3 in TA patients**

	**Erythrocyte sedimentation rate, mm/h**	**C-reactive protein, mg/l**	**Pentraxin-3, ng/ml**
Class of vascular involvement			
1 (n = 4)	28, 34 (4 to 44)	8.3, 6.0 (2.7 to 19)	5.5, 6.2 (2.9 to 6.7)
2A (n = 4)	15, 17 (7 to 22)	3.0, 1.0 (0.1 to 11)	15, 6.8 (6.2 to 44)
2B (n = 3)	33, 33 (29 to 38)	17, 12 (1.2 to 37)	5.3, 5.0 (3.9 to 7.0)
3 (n = 1)	4.0	2.0	6.3
4 (n = 0)	NA	NA	NA
5 (n = 30)	21, 15 (1 to 78)	5.5, 1.9 (0.1 to 40)	8.2, 5.1 (3.0 to 5.1)
Presence of aneurysms (n = 16)	23, 15 (1 to 78)	5.7, 2.7 (0.3 to 40)	6.6, 5.8 (1.3 to 23)
Absence of aneurysms (n = 26)	20, 16 (2 to 68)	6.9, 1.2 (0.1 to 37)	11, 5.0 (2.2 to 55)
Active (NIH criteria n = 12)	34, 38 (3 to 73)	12, 8.6 (0.3 to 40)	12, 6.9 (2.2 to 55)
Inactive (NIH criteria n = 30)	16, 12 (1 to 78)	3.5, 1.9 (0.1 to 19)	7.1, 5.0 (1.3 to 44)

Results are presented as mean, median (range). NA, not applicable; NIH, National Institutes of Health.

**Figure 3:**
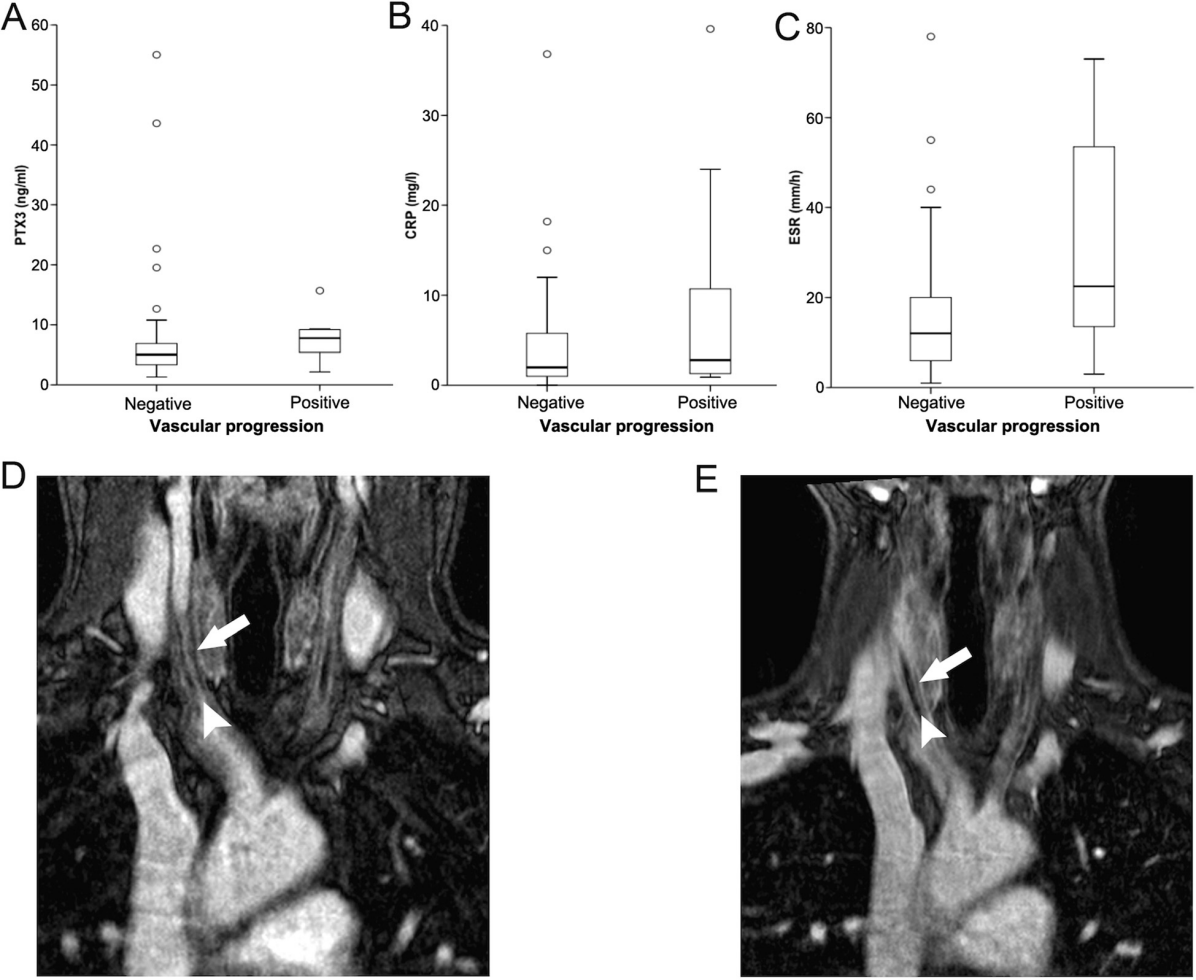
**Pentraxin-3 (PTX3) levels and progressive vascular involvement in Takayasu arteritis (TA). (A-** **C)** PTX3 and C-reactive protein (CRP) concentration and erythrocyte sedimentation rate (ESR) levels in patients with TA stratified according to the presence of progression of vascular wall involvement on longitudinal imaging follow up. **(D,** **E)** Representative images show progression of vascular wall involvement between May 2012 **(D)** and May 2013 **(E)**. Wall thickness in the right carotid artery changed from 1.7 mm in May 2012 to 2.7 mm in May 2013 (white arrows); arterial lumen was patent and stenotic in May 2012, and was occluded in May 2013 (white arrowheads).

The scenario changes when patients are stratified based on the presence of vascular inflammation, assessed by the presence of vascular wall enhancement after contrast media infusion (Figure 2G-I). Concentrations of PTX3 were higher in patients with wall enhancement (median 16 ng/ml (range 6.2 to 55 ng/ml) versus 5.2 ng/ml (range 2.5 to 44 ng/ml), *P* = 0.016). CRP concentrations and ESR were similar in the two groups. Neither marker discriminated patients with or without evidence of progression of arterial lesions at longitudinal imaging follow-up (Figure 3A-C).

### Anti-cytokine therapy influences the ability of PTX3 to identify vascular inflammation and progression in TA

**Figure 4:**
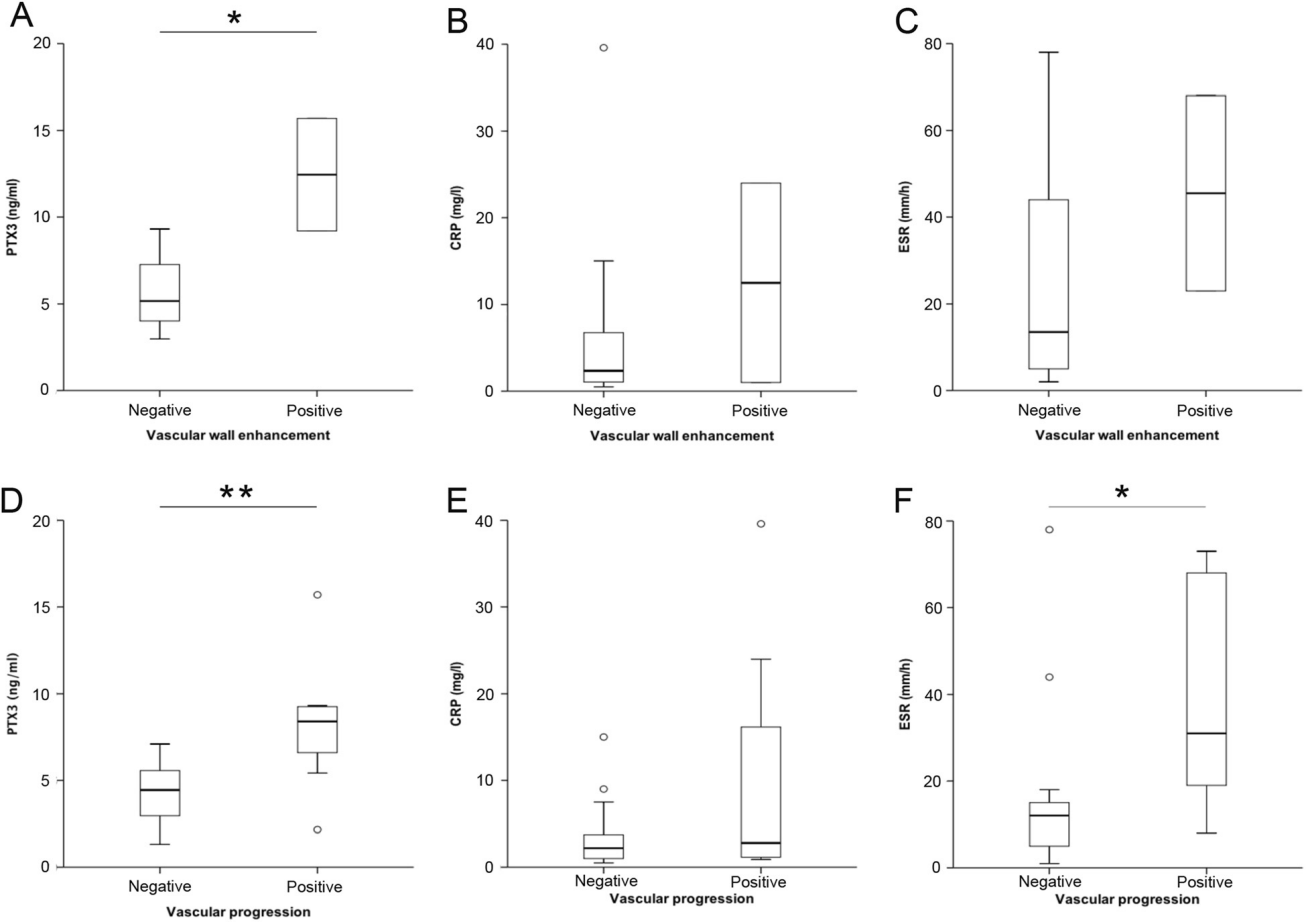
**Anti-cytokine treatment interferes with the performance of Pentraxin-3 (PTX3) as a biomarker in Takayasu arteritis (TA). (A**-**C)** Plasma PTX3, C-reactive protein (CRP) and erythrocyte sedimentation rate (ESR) levels in patients with TA without anti-cytokine therapy, stratified according to the presence of vascular wall enhancement after contrast medium infusion. **(D**-**F)** Concentrations of PTX3, CRP and ESR in patients with TA without anti-cytokine therapy, stratified according to the progression of vascular wall involvement on longitudinal imaging follow up.

Eighteen of the forty-two patients (43%) were treated with TNF-α or IL-6-receptor inhibitors. Inflammatory cytokines are known inducers of PTX3 expression and release. Indeed, the median PTX3 concentration of TA patients with arterial wall enhancement who were not receiving TNF-α or IL-6-receptor inhibitors (n = 24) was 12 ng/ml (range 9.2 to 15 ng/ml) versus 5.2 ng/ml (range 3.0 to 9.3 ng/ml) in those without wall enhancement (*P* = 0.044, Figure 4A). In contrast CRP levels or ESR were similar regardless of imaging evidence of vessel wall inflammation (Figure 4B, C). In the absence of anti-cytokine therapies, PTX3 concentrations were higher in TA patients with vascular progression than in stable patients (median 8.4 ng/ml (range 2.2 to 16 ng/ml) versus 4.4 ng/ml (range 1.3 to 7.1 ng/ml), *P* = 0.010, Figure 4D). CRP levels were similar in patients with and without vascular progression (Figure 4E) while ESR was higher in patients undergoing progression (median 31 mm/h (range 8 to 73 mm/h) versus 11 mm/h (range 1 to 78 mm/h), *P* = 0.036, Figure 4F).

## Discussion

Here we show that plasmatic PTX3 in patients with TA reflect specific features of vessel involvement of TA, differently from CRP and ESR. This might depend on the specific feature of PTX3, which is locally produced at sites of inflammation and plays an emerging role in vessel biology, and whose generation is possibly elicited in response to stimuli distinct from those that trigger the production of other pentraxins, such as CRP [12,13,15,16,18-20,26-28]. PTX3 is involved in the maintenance of vascular homeostasis: it plays a protective role in various vessel diseases in which inflammation and vascular remodelling both contribute to the clinical outcome, such as atherosclerosis [29] or the response to balloon angioplasty [30]. PTX3 is a novel and informative marker of cardiovascular risk and of atherosclerotic plaque inflammation, which possibly reflects diverse facets of the inflammatory response from commonly used acute phase reactants [31-33].

Our results confirm that CRP and ESR reveal the systemic inflammatory burden associated with TA. However, these markers apparently fail to provide information on the events that take place specifically within the inflamed vessel walls: their variations do not correlate with the presence of vascular inflammation as assessed by the presence of contrast enhancement and the progression of the vascular wall involvement. In the subgroup of patients not treated with anti-cytokine therapy, ESR was higher in patient swith vascular progression. This correlation was not evident in patients treated with biologic agents. Recent data indicate that vascular inflammation and progression occur even after ESR and CRP levels have abated upon the pharmacological blockade of inflammatory cytokines [1,34,35] suggesting that vascular inflammation in TA depends on multiple pathways, some of which remain strictly localized within the vascular lesions and do not result in an acute-phase response [10,11]. Moreover, the relationship between vascular progression and inflammation is still elusive, as various non-overlapping inflammatory pathways sustain vascular remodelling [10,11,36-39].

Recent studies have shown that PTX3 blood levels are increased in TA and suggest that it might be generated within the inflamed vessel wall, possibly by *vasa vasorum* endothelial cells or macrophages within the inflammatory infiltrate [18-20]. These studies observed a correlation between plasma PTX3 concentration and activity in TA. Our results are consistent with significant correlation between variations of systemic PTX3 levels and the presence of enhancement within arterial lesions on imaging assessment. PTX3 identified vascular progression only in patients who were not being treated with anti-cytokine agents. This is in agreement with the observation that several inflammatory cytokines, and TNF-α in particular, foster PTX3 expression and release [12,40]. IL-6 is not known to directly induce PTX3 expression and release [27,40]. However, it could induce the generation of TNF-α and IL-1β, thus, indirectly modulating PTX3 levels.

NIH criteria have a high sensitivity to systemic inflammation in TA [5], and we did not observe correlation between plasma PTX3 and TA activity evaluated with these criteria. Previous studies that evaluated plasma PTX3 concentration in TA defined disease activity with other, *ad hoc*-created criteria that more accurately reflect arterial involvement [18-20]. One of these studies was performed at our institution before the widespread use of biologic agents [18]. It is possible that the correlation between plasma PTX3 and TA activity observed in previous reports could be due to a smaller fraction of patients receiving anti-cytokine therapy and by using definitions of disease activity that are less influenced by systemic inflammation than NIH criteria [18]. These results reinforce the hypothesis that the progression of arterial involvement in TA depends on a variety of mechanisms, including remodelling as well as inflammation of the arterial wall, which sometimes causes the acute-phase response and systemic inflammatory symptoms.

Our study has limitations: first it was an observational cross-sectional study. Second, TA is a rare disease and large cohorts are difficult to establish. The relatively small number of patients might have limited the statistical power of the study. Finally, we did not use histology as a reference for assessing vascular inflammation. However, surgery is quite rarely performed in TA, and to obtain arterial specimens to confirm the results of imaging is difficult. MRI can reveal oedema or contrast enhancement within the thickened arterial wall, which might be associated with ongoing inflammation. Contrasting results are reported in the MRI assessment of TA activity as assessed by NIH criteria [24,41]. However, NIH criteria could not accurately reflect arterial inflammation and progression [5,7]. Thus, although there is no consensus on the preferred mode and the protocol for the assessment of arterial inflammation [42], imaging currently remains the only practical way to study local vessel inflammation in TA.

## Conclusions

Unlike CRP and ESR, plasma PTX3 appears to be scarcely influenced by overt and systemic inflammation in TA. In contrast, PTX3 levels are increased in the presence of vascular wall enhancement, which could reflect ongoing arterial inflammation. In the absence of anti-cytokine therapy, plasma PTX3 is also significantly higher in patients experiencing longitudinal disease progression. PTX3 may thus be used as a biomarker of actual arteritis in TA.

## Abbreviations

CRP: C-reactive protein
CTA: computer tomography angiography
ESR: erythrocyte sedimentation rate
FFD: Fast Field Echo
HR: High Resolution
IL: interleukin
MRA: magnetic resonance angiography
NIH: National Institutes of Health
PD: Proton Density
PTX3: Pentraxin-3
SLE: systemic lupus erythematous
TA: Takayasu arteritis
TE: Time Echo
TNF: tumour necrosis factor
TR: Time Repetition
TSE: Turbo spin Echo
US: ultrasonography
